# Research on Safety of Aero-Engine Oil Pipe Under Heating Conditions Based on Fluid–Solid Thermal Coupling

**DOI:** 10.3390/ma17215137

**Published:** 2024-10-22

**Authors:** Yuepeng Yang, Fang Wang, Fang Wen, Jie Jin

**Affiliations:** 1 School of Energy and Power Engineering, Beihang University (BUAA), Beijing 100191, China; sy2204511@buaa.edu.cn (Y.Y.); fwang@buaa.edu.cn (F.W.); by2104508@buaa.du.cn (F.W.); 2 Jiangxi Research Institute, Beihang University (BUAA), Nanchang 330096, China; 3 Chengdu Innovation Research Institute on Aircraft Power, Beihang University (BUAA), Chengdu 611930, China

**Keywords:** aero-engine oil pipe, standard document, aviation kerosene, fluid–solid thermal coupling, pipe safety

## Abstract

This paper examines the safety of aero-engine pipelines under different heating conditions. Based on the fire test standard documents, a model of an aero-engine oil pipe was constructed, and its safety under heating conditions that meet the standard was analyzed using fluid–solid thermal coupling. The pipe material was stainless steel 1Cr18Ni9Ti, and the oil inside the pipeline was China RP-3 kerosene. To simulate the different working conditions or pump failure scenarios, various kerosene inlet flow rates were used for the calculations. The results indicate that the pipe wall exhibits an uneven temperature distribution under standard heating conditions. As the kerosene flow rate decreases, the pipe wall temperature rises, and heat transfer deterioration occurs. The increase in the pipe wall temperature reduces the material’s strength, while the uneven temperature distribution generates thermal stress, further increasing the safety risk. When the kerosene flow rate is reduced to a certain level, the equivalent stress in the pipe wall exceeds the material’s yield strength, leading to a high risk of rupture.

## 1. Introduction

Aircraft misfire is one of the most common and dangerous accidents that occurs during the operation and maintenance of aircraft. The engine, which operates in a high-temperature environment, poses significant potential hazards due to the use of flammable organic substances, such as aviation kerosene and lubricants [1]. If a pipe ruptures under hot conditions and leaks, the oil will be ignited at high temperatures, causing a secondary fire. According to Clause 33.17 of the Aviation Engine Airworthiness Regulations [2] issued by the Civil Aviation Administration of China (CAAC) on 30 January 2011, all external pipelines, fittings, and other components that carry flammable liquids during normal engine operation must be verified by the CAAC to be fire resistant or fireproof. Similarly, the U.S. Federal Aviation Regulations (FAR) stipulate that the key components of aircraft engines, including pipelines, must remain operational during a misfire to ensure safe engine shutdown [3]. The European Union Aviation Safety Agency (EASA) also mandates fire prevention and resistance measures for engines in its airworthiness regulation CS-E [4].

Fire testing is the most common and effective method for verifying the fireproofing of pipes. Current academic research on aero-engine pipeline fire prevention and fire tests mainly focuses on the combustor [5,6,7,8] used in testing and the establishment of fire prevention regulations [9,10]. Wu discussed the three main factors influencing engine fire testing, based on fire tests of aero-engine components, such as bent pipes, connectors, and fuel tanks [11]. Jiang et al. focused on the operational conditions of pipeline connectors under fire testing conditions, analyzing the effects of the flow rate, pressure, and inlet temperature [12]. Wang X. proposed a method to assist in assessing the fire resistance of pipelines based on the pipe wall temperature outside the fire zone [13]. Wang W. designed a fire test system for pipe assemblies based on regulatory requirements [14]. Although there has been considerable research on the fire testing of engine pipelines, few studies have specifically addressed the safety of the pipe wall under heated conditions, including the pipe wall temperature and stress distribution.

Wang found instances of pipe wall rupture and failure during fire tests on engine pipelines [15]. The oil pressure within an engine’s oil pipeline is high, creating significant stress, particularly in the circumferential direction. Under elevated temperatures, excessive wall temperatures can substantially reduce pipe strength, leading to potential safety issues. This is the primary cause of failure for an engine’s oil pipeline under heated conditions, and it is necessary to conduct an in-depth study of this issue.

Fire tests are generally conducted under conditions of high flow rates within a pipe, which allow the oil to carry away sufficient heat and keep the wall temperature from becoming excessively high. However, it is challenging to perform tests under low flow conditions. If a pipe ruptures while being heated by flames, the high-pressure oil inside will spray out and ignite, potentially leading to serious accidents, such as fires on the test bench. Therefore, conducting fire tests to study the safety of aero-engine pipe walls under low flow conditions involves a high level of risk.

To investigate the safety of aviation engine oil pipelines under heated conditions at various flow rates, including lower flow rates, and to provide a reference for subsequent low-flow pipeline fire tests, this paper used the aviation industry standard “Civil Aircraft Hose and Pipe Assembly Fire Test Requirements” (HB 7044-2014) [16] and other relevant documents to build a model of an aero-engine oil pipe. Utilizing the numerical simulation software ANSYS Fluent 2023R1, numerical simulations were performed to analyze the fluid–solid thermal coupling of the oil pipe under different testing conditions. The flow calculations provided the pressure and temperature distribution within the pipeline, upon which stress calculations were conducted, and the safety was assessed by comparing the stress levels with the material strength. This study aimed to provide valuable insights for practical applications, including the fire testing, design, and thermal protection of engine pipes.

## 2. Computational Models, Methods, and Mesh

### 2.1. Computational Domain Models

**Figure 1:**
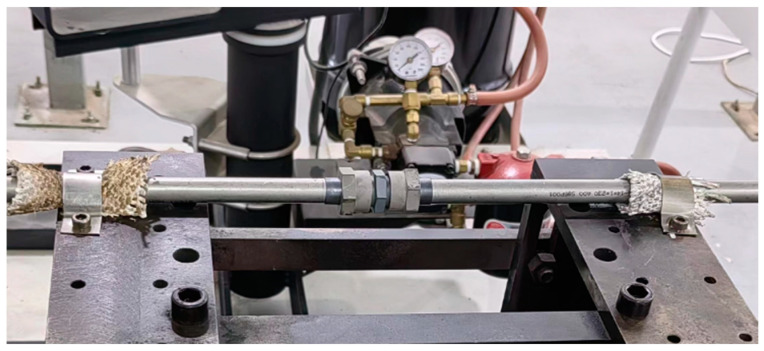
Pipe assembly for flareless connection, swager type, 28 MPa.

The physical model of the computational domain, primarily consisting of three regions—the in-pipe oil, the pipe, and the out-pipe fluid—was constructed using the ANSYS Design Modeler. The oil-through pipe features straight sections at both ends and a connecting section in the middle, modeled in accordance with aviation industry standards HB 7044-2014 and HB 7842-2008 [17]. The outer diameter of the straight pipe is 16 mm and the thickness is 1.5 mm. As shown in Figure 1, the connecting section contains components such as the pipe sleeve, the outer sleeve nut, the end of the joint, etc. Since the coupling heat transfer of the pipe with the fluid is a primary focus of the flow heat transfer calculations, the thermal resistances are superimposed based on the dimensions of each component given in the corresponding standard documents; thus, the connecting section is equivalent to a cylinder with a length of 58 mm, an outer diameter of 20.5 mm, and an inner diameter of 13 mm. The total length of the pipe, including the connecting section, is 600 mm.

**Figure 2:**
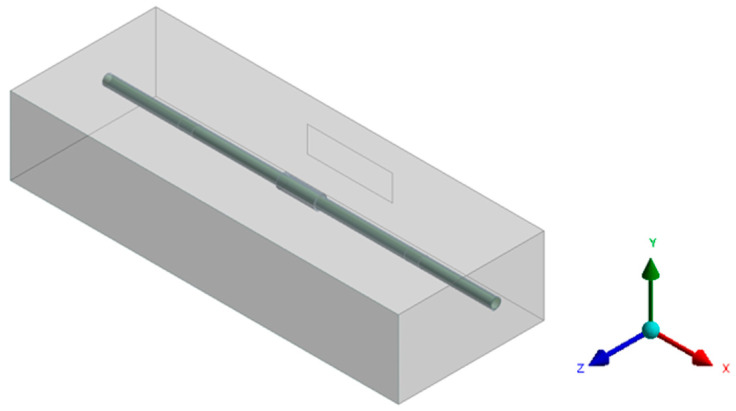
Geometric model of computational domain.

The geometric model of the computational domain is depicted in Figure 2. The fluid domain surrounding the pipe is a cube measuring 600 mm in length, 225 mm in depth, and 120 mm in height. One face of the cube features a square inlet at its center, measuring 132 mm wide and 40 mm high, with the pipe positioned 100 mm directly opposite the inlet. In order to simplify the calculation, standard high-temperature flue gas is utilized instead of gas produced by a combustor. The flue gas is injected into the computational domain through the square inlet to heat the pipe, and the dimensions of the gas flow inlet meet the standard requirement of a minimum pipe length of 128 mm for encircled heating. In this model, the positive direction of the *X*-axis corresponds to the fluid flow direction within the pipe, the *Y*-axis is aligned parallel to the gravitational direction, and the positive *Z*-axis represents the flow direction of the high-temperature flue gas.

### 2.2. Pipe Materials and Fluid

According to the standard document, the pipe material is stainless steel 1Cr18Ni9Ti. This alloy is classified as a general-purpose chromium–nickel austenitic stainless steel, known for its excellent ductility and toughness, and has one of the highest yield strengths among austenitic stainless steels. 1Cr18Ni9Ti is widely employed in the aviation, aerospace, chemical, and medical industries. Its chemical composition is presented in Table 1.

According to the China Aviation Materials Handbook [18], the density of 1Cr18Ni9Ti is 7900 kg/m^3^, its specific heat is 0.502 kJ/(kg·K), and its thermal conductivity is shown in Table 2. By importing the above physical properties into the numerical simulation software, the modeling of the pipe can be completed, facilitating the subsequent fluid–solid thermal coupling calculations.

**Figure 3:**
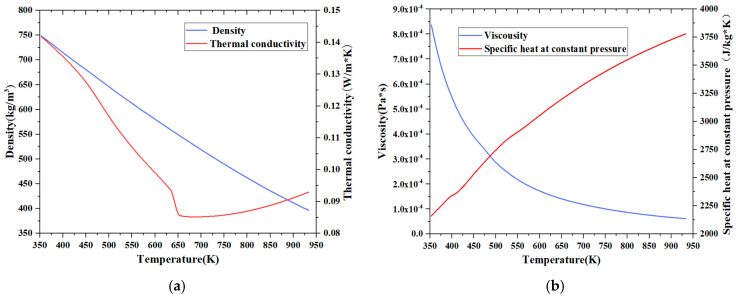
Thermophysical properties of RP-3 aviation kerosene at 28 MPa: (**a**) density and thermal conductivity; (**b**) viscousity and specific heat.

The fluid domain inside the pipe is 600 mm long and 13 mm in diameter. In this paper, China RP-3 aviation kerosene is utilized as the working fluid in the pipe. According to the working conditions of the pipe, the fluid pressure is 28 MPa. Considering that the physical properties of RP-3 aviation kerosene change drastically with an increase in the temperature when it is above the critical pressure, this study adopts the 10-component substitution model [19] to simulate the thermal properties of RP-3 aviation kerosene, based on the generalized corresponding state rule and the Peng–Robinson equation [20]. According to the simulation results for the 10-component model, the critical pressure of RP-3 aviation kerosene is 2.4 MPa [21], which is basically consistent with the experimental measurements. The calculated thermophysical parameters of RP-3 aviation kerosene at 28 MPa are shown in Figure 3. It is evident that the parameters, including density, constant-pressure specific heat, thermal conductivity, and viscosity, exhibit significant variations with increasing temperature. Compared to normal fluids, the flow heat transfer mechanism between the supercritical kerosene and the pipe wall becomes notably complex due to these substantial property changes [22,23]. Therefore, when analyzing the heat transfer, it is essential to account for the variations in the physical properties of the kerosene as its temperature changes.

### 2.3. Computational Methods

The coupled flow–solid–heat calculations were conducted by discretizing and solving the Navier–Stokes equations using the finite volume method, achieving second-order accuracy for each discretization format. The RNG k-ε two-equation model was employed to address turbulent flow, with the near-wall region treated using an enhanced wall treatment method that integrates a two-layer model with enhanced wall functions. The Wolfstein one-equation model was applied to resolve the near-wall low-Reynolds-number region, while the RNG k-ε equations were used to handle the fully developed turbulent region. The combination of the RNG k-ε model with enhanced wall functions is widely regarded as the standard approach for the numerical simulation of flow and heat transfer for supercritical kerosene [24,25]. Pressure–velocity coupling was implemented using the coupled algorithm. For the structural calculations, a finite element approach (FEA) was employed, which is the more common method when using numerical simulations to analyze the safety of pipelines, such as determining whether they will rupture or not [26].

### 2.4. Validation of the Computational Method

**Figure 4:**
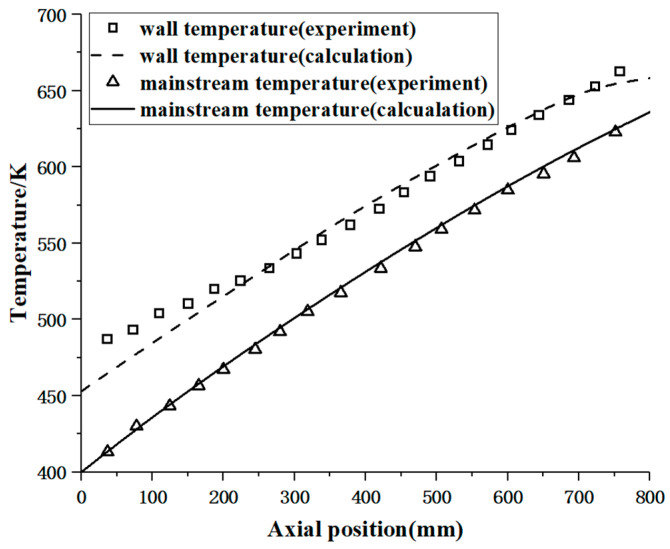
Validation of the computational method.

To validate the credibility of the aforementioned computational methods, a numerical simulation was performed based on the convective heat transfer experiments conducted in a circular tube with supercritical RP-3 aviation kerosene, as reported in reference [27]. The pipe had an inner diameter of 1 mm and a length of 800 mm, with the kerosene maintained at a pressure of 4 MPa. The inlet mass flow rate was G = 2546.48 kg/(m^2^·s), and the experimental setup utilized electrical heating with a heat flux density of 561 kW/m^2^. Figure 4 presents the temperature distributions along the tube wall and the mainstream temperatures obtained from both the experiments and the numerical calculations. The results indicate a good agreement between the numerical predictions and the experimental data, demonstrating that the numerical computation method employed possesses a high degree of credibility.

### 2.5. Mesh

**Figure 5:**
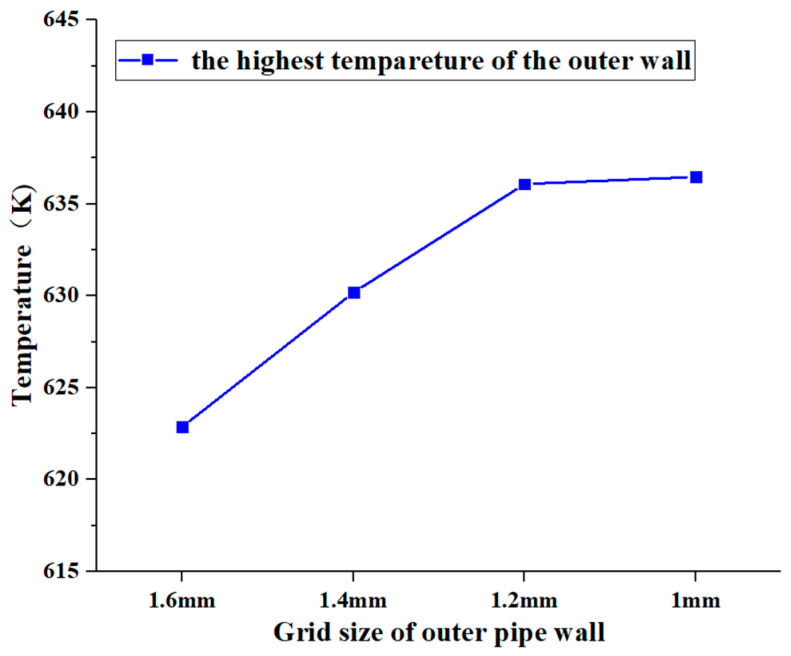
Grid independence verification.

**Figure 6:**
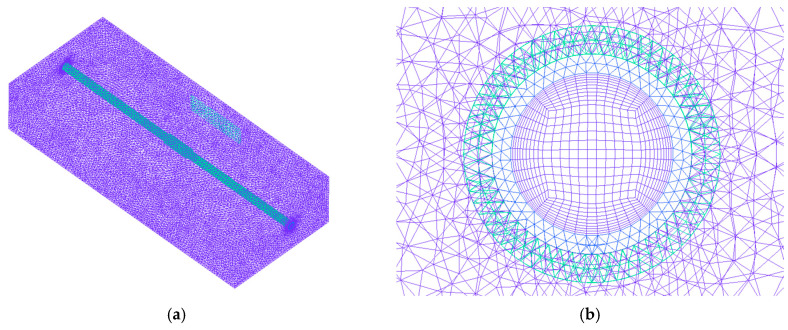
Computational domain grid: (**a**) isometric; (**b**) local.

The fluid domain inside the pipe employed a hexahedral structured grid, with the first grid layer positioned 0.1 mm from the pipe wall. Conversely, a tetrahedral unstructured grid was utilized within the pipe itself and the exterior fluid domain. The grid cell sizes on the outer tube wall were set at 1 mm, 1.2 mm, 1.4 mm, and 1.6 mm, respectively. The highest outer wall temperature served as the metric for grid independence verification under a flow rate of 5.07 L/min within the heated pipe. Figure 5 illustrates the results of this verification. As the grid cell size on the outer wall is refined, there is an observed increase in the maximum wall temperature. However, once the cell size reaches 1.2 mm, further refinement no longer increases the maximum wall temperature. Therefore, this grid configuration, consisting of 2.21 million cells, was selected for the subsequent numerical simulations in this study. The grid is illustrated in Figure 6.

## 3. Fluid–Solid Thermal Coupling Calculations

### 3.1. Boundary and Working Conditions

In this paper, numerical simulations of the flow–solid heat coupling are conducted for the aero-engine through-oil pipeline and its connecting sections under standard heating conditions. Considering the effects of gravity, the computational conditions are as follows.

The inlet pressure of the RP-3 aviation kerosene is set at p_oil_ = 28 MPa, with an inlet temperature of T_in_ = 366.15 K (93 °C). The inlet flow rate of the kerosene is 5.07 L/min. Additionally, to simulate the scenario of reduced flow rates under different operating conditions or due to pump failure, five conditions with reduced flow rates are established: 3.80 L/min, 2.54 L/min, 1.27 L/min, 0.50 L/min, and 0.25 L/min. The kerosene inlet conditions are summarized in Table 3 below.

The external hot flue gas inlet pressure is set at p_gas_ = 0.1 MPa, with an inlet temperature of T_gas_ = 1363.15 K (1090 °C) and an inlet velocity of 21 m/s. Under these conditions, when the kerosene flow rate is 5.07 L/min, the steady-state heat transfer power is 1370 W, meeting the standard requirements. The ambient temperature is 300 K.

### 3.2. Analysis of Calculation Results under 5.07 L/Min Flow Condition

**Figure 7:**
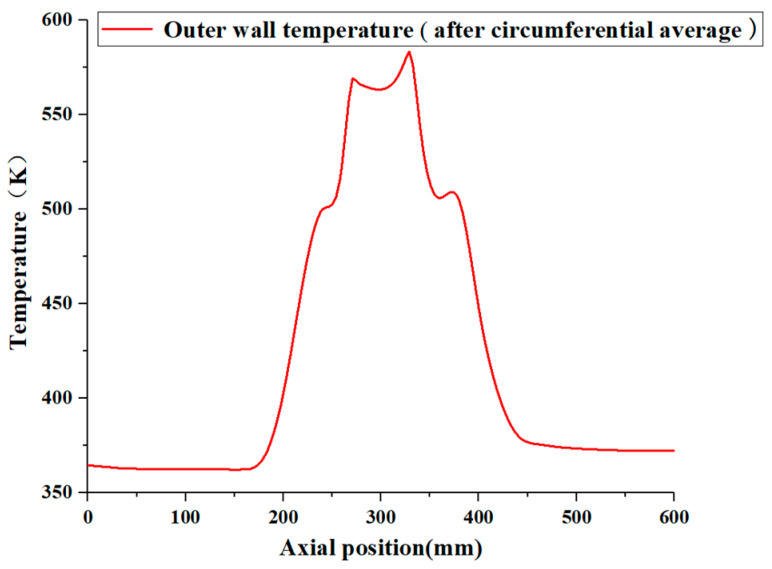
The axial distribution of the pipe wall temperature at a 5.07 L/min kerosene flow rate.

Figure 7 shows the axial distribution of the outer wall temperature of the pipe after averaging in the circumferential direction when the kerosene inlet volume flow rate is 5.07 L/min. The figure reveals a ‘three-layer stepped’ distribution of the wall temperature along the axial direction, characterized by higher temperatures in the middle and lower temperatures on the sides. The middle section of the pipe is primarily subjected to the lateral flow of the high-temperature flue gas, resulting in intense forced convection heat transfer at the pipe wall, which leads to elevated wall temperatures. At an axial position of approximately 170 mm, the pipe wall begins to make contact with the hot flue gas, leading to a sharp increase in the temperature. Subsequently, as the temperature difference between the outer pipe wall and the hot flue gas diminishes, the heat transfer is reduced, and the rate of increase in the wall temperature slows. Near the connecting section (at an axial position of about 260 mm), the wall temperature exhibits a significant rise once again. This phenomenon can be attributed to the thickening of the pipe wall at this location, which introduces an annular heating plane that increases the heating surface area, consequently leading to a significant rise in the wall temperature. The highest temperature of the ‘second layer’ is observed at an axial position of about 370 mm, which is noteworthy due to the higher risk of rupture due to the thinner wall and higher temperature at this location. Heat transfer on both sides of the pipe is primarily governed by axial heat conduction through the wall and natural convection with the surrounding air, resulting in a weak heat transfer intensity and lower wall temperatures. Since the ambient temperature is lower than the wall temperature, the wall temperature gradually decreases in the axial direction, particularly in areas less influenced by the hot flue gas.

**Figure 8:**
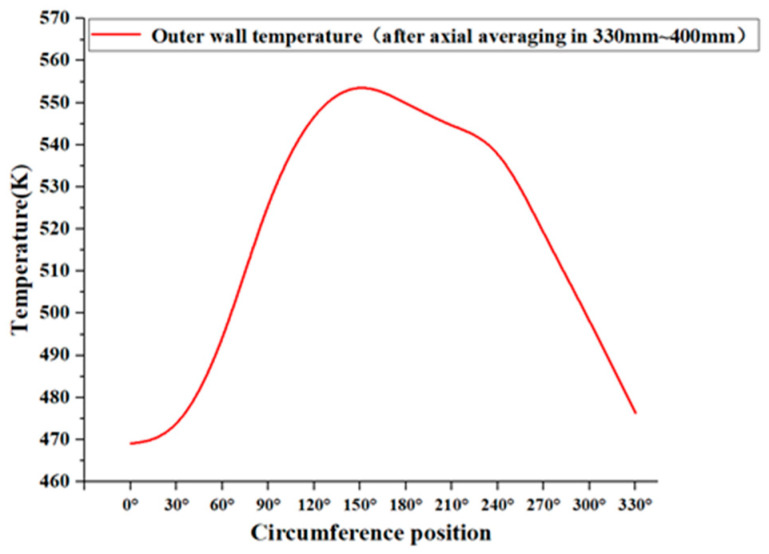
The circumference distribution of the pipe wall temperature at a 5.07 L/min kerosene flow rate.

Figure 8 illustrates the circumferential distribution of the pipe wall temperature, averaged axially from 330 mm to 400 mm, with the kerosene inlet flow rate set at 5.07 L/min. The Z-direction is defined as 0°, and rotation towards the *Y*-axis is considered the positive direction. The figure indicates that the wall temperature on the side of the pipe facing the flue gas outlet (90° to 270°) is higher than that on the side opposite the flue gas. Furthermore, the outer wall temperature on the upper side of the pipe facing the flue gas outlet (90° to 180°) exceeds that on the lower side, with the maximum wall temperature occurring at approximately 150°. This phenomenon can be attributed to the lower density of the flue gas compared to that of air, causing it to ‘lift’ in the flow, which results in a slightly enhanced heat transfer intensity on the upper side of the outer wall compared to the lower side. Given that the increase in temperature leads to a decrease in material strength, it is essential to account for the temperature and strength characteristics of the upper side of the pipe wall in subsequent safety analyses.

Under heated conditions, the wall temperature of the pipe exhibits an uneven distribution in all directions. This uneven temperature distribution leads to thermal stress, which can further deteriorate the working environment of the pipe and elevate the risk of wall rupture.

### 3.3. Effect of Kerosene Inlet Flow on Convective Heat Transfer

**Figure 9:**
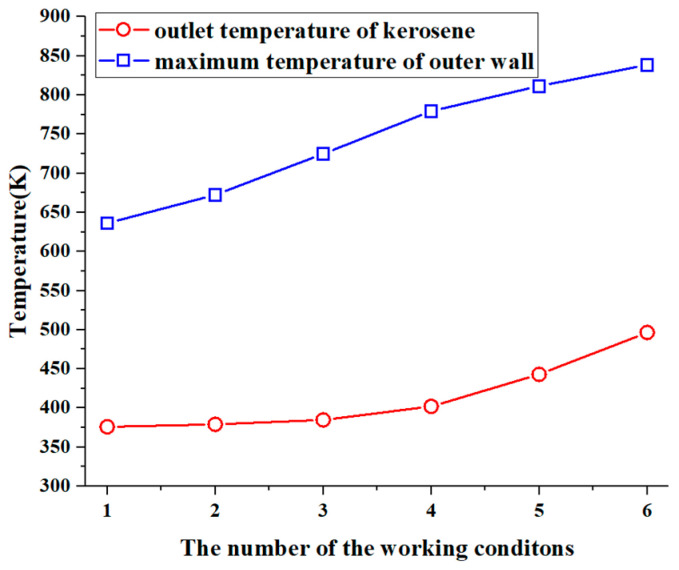
The maximum temperature of the outer wall and the outlet temperature of the kerosene.

Figure 9 illustrates the maximum temperature of the outer wall of the pipe and the outlet temperature of the RP-3 aviation kerosene when the heat transfer reaches a steady state at different inlet flow rates. It is evident that both the outer wall temperature of the pipe and the outlet kerosene temperature increase as the flow rate decreases, indicating a deterioration in the heat transfer within the pipe. When the inlet flow rate is reduced to 0.25 L/min, the maximum temperature of the outer wall rises by approximately 200 K compared to the inlet flow rate of 5.07 L/min. Such a significant temperature increase leads to a substantial reduction in the yield strength, tensile strength, and other mechanical properties of the pipe material, greatly increasing the likelihood of a wall rupture. The stress and strength analysis of the pipe under these working conditions will be addressed in the next section.

**Figure 10:**
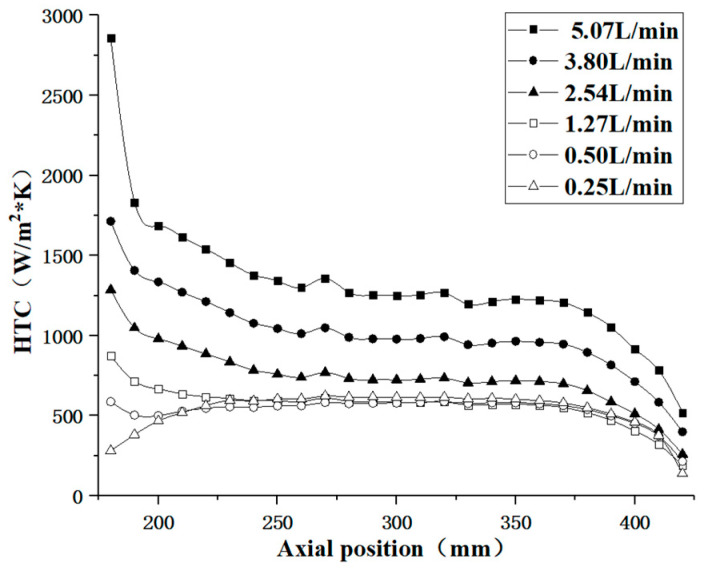
Convective heat transfer coefficient along the pipe under different kerosene inlet volume flows.

Figure 10 illustrates the distribution of the convective heat transfer coefficient (HTC) within the pipe at various inlet flow rates in the region significantly affected by the hot flue gas (axial positions from 180 mm to 420 mm). The figure demonstrates that when the kerosene inlet flow rate is higher and the kerosene temperature remains relatively low, the thermal physical properties of the kerosene do not undergo significant changes, resulting in a convective heat transfer coefficient that decreases along the flow direction. In the inlet section of the pipe, the wall temperature rises rapidly due to the influence of the hot flue gas, leading to a more intense heat transfer; however, the convective heat transfer coefficient exhibits a downward trend because the increase in the kerosene temperature is relatively slow. Near the outlet side, as the influence of the hot flue gas diminishes, the pipe wall temperature begins to decrease along the flow direction, resulting in a weakened heat transfer.

As the kerosene inlet flow rate decreases, the convective heat transfer coefficient also diminishes. When the flow rate drops below 1.27 L/min, the convective heat transfer coefficient exhibits minimal change with further reductions in the flow rate, which diverges from the heat transfer behavior typically observed in constant-property fluids. As illustrated in Figure 3 and Figure 9, there is a significant temperature rise in the kerosene within the pipe under these operating conditions, indicating substantial changes in its thermal physical properties. Therefore, it is essential to consider the effects of variations in the thermophysical properties of the kerosene when further analyzing the convective heat transfer between the kerosene and the pipe wall.

**Figure 11:**
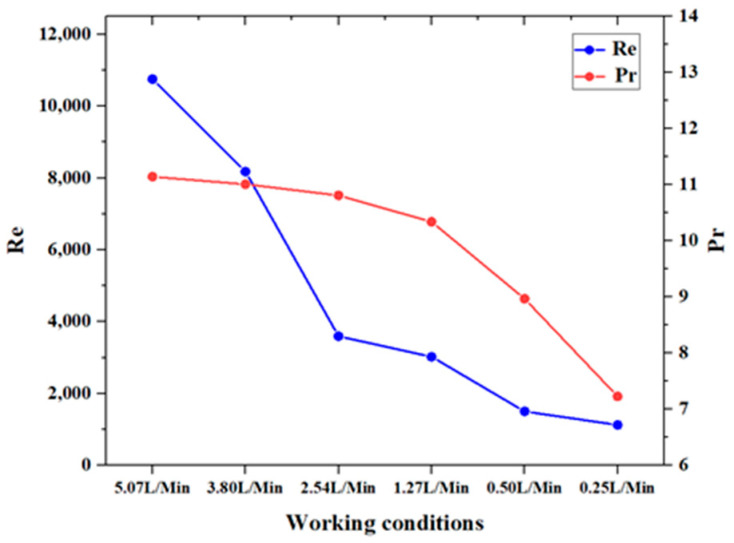
Reynolds (Re) and Prandtl (Pr) numbers at an axial position of 300 mm under different working conditions.

**Figure 12:**
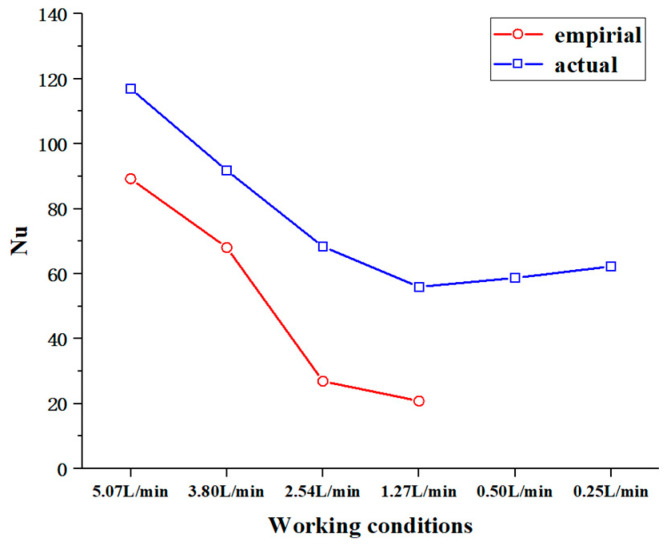
Actual and empirical Nusselt numbers at an axial position of 300 mm under different working conditions.

Figure 11 presents the averaged Reynolds number (Re) and Prandtl number (Pr) at the mid-section of the pipe (averaged over 200–400 mm) under different working conditions. The figure shows that as the inlet flow rate decreases, there are significant changes in both the Re and Pr, indicating notable variations in the flow characteristics and thermophysical properties of the kerosene. Figure 12 illustrates the actual Nusselt number (Nu) calculated at this position compared to the Nusselt number obtained from the empirical correlation. The empirical correlation used is the Gnielinski heat transfer correlation (this relation does not apply to working condition 5 and 6 due to the Reynolds number), and is based on the heat transfer behavior of constant-property fluids [28].

As can be seen from the figure, the actual Nusselt numbers are significantly higher than those predicted by the empirical correlation, indicating that the heat transfer behavior of supercritical aviation kerosene differs from that of constant-property fluids. The variation in the thermophysical properties enhances heat transfer. As the inlet flow rate decreases, the Nusselt number shows a downward trend. However, when the flow rate drops below 1.27 L/min, the Nusselt number increases, suggesting that the changes in the thermophysical properties have a pronounced effect on the heat transfer behavior of the kerosene.

## 4. Pipe Stress Calculation and Safety Analysis

Based on the results of the flow and heat transfer calculations, ANSYS Mechanical 2022R1 was employed to calculate the stress on the pipe. The pressure of the kerosene acting on the pipe and the temperature distribution of the pipe wall were applied to the coupling interface and pipe wall via interpolation. In accordance with the fire test setup, normal constraints were applied to the pipe wall at axial positions of 120–140 mm and 460–480 mm, while fixed constraints were applied to one end of the pipe to restrict axial and tangential sliding.

**Figure 13:**
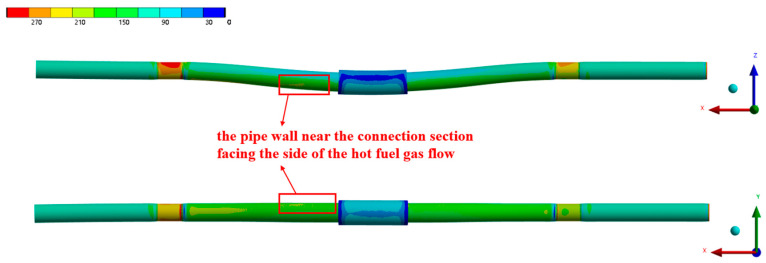
The equivalent stress distribution of the pipe under the working condition of 5.07 L/min.

Figure 13 presents the equivalent stress distribution of the pipe under heated conditions, with an inlet kerosene flow rate of 5.07 L/min. The figure shows that, due to the pressure of the kerosene, the pipe wall experiences circumferential stress, causing uniform expansion in the circumferential direction, while the thermal stress resulting from the uneven temperature distribution across the pipe wall leads to non-uniform expansion in all directions. Under the combined effects of the kerosene pressure, wall temperature distribution, and applied constraints, the pipe wall expands in the direction of the incoming flue gas flow. Ignoring the stress concentrations near the constraint locations, the section of the pipe wall near the connection, particularly the upper side facing the hot gas flow (the circumferential position of 150°), experiences the highest stress.

**Figure 14:**
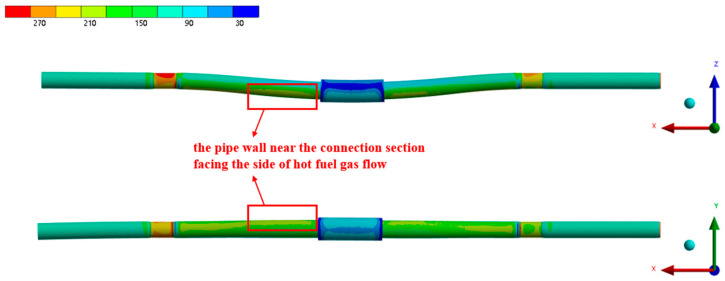
The equivalent stress distribution of the pipe under the working condition of 3.80 L/min.

**Figure 15:**
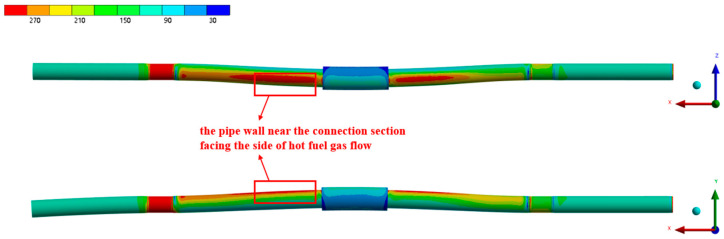
The equivalent stress distribution of the pipe under the working condition of 0.50 L/min.

Figure 14 and Figure 15 present the equivalent stress distribution of the pipe wall at inlet flow rates of 3.80 L/min and 0.50 L/min, respectively. The figures demonstrate that the overall stress distribution of the pipe wall remains largely consistent with the results for a flow rate of 5.07 L/min. However, as the kerosene inlet flow rate decreases, there is a noticeable increase in the equivalent stress values. This increase is attributed to the larger temperature gradient across the pipe wall, which leads to a corresponding rise in the thermal stress. The decrease in the flow rate also causes an increase in the pipe wall temperature, resulting in a reduction in the material strength and a significantly increased risk of pipe wall rupture.

**Figure 16:**
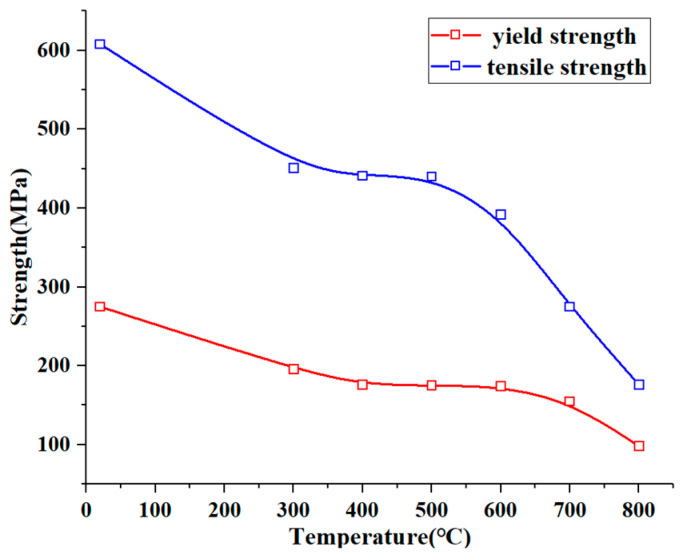
The fitting curve of the relationship between material strength and temperature.

According to the Materials Handbook, the yield strength of a pipe’s wall material decreases as the wall temperature increases, making the regions with higher temperatures and elevated equivalent stress more susceptible to rupture. To establish the relationship between the material strength and temperature (°C), the yield strength and tensile strength of 1Cr18Ni9Ti at various temperatures were polynomially fitted, as shown in Figure 16. The corresponding fitting formula is as follows:

σ_0.2_ = 286.06034 − 0.555846746 ∗ t + 0.0010096479 ∗ t^2^ − 0.0000006787759596 ∗ t^3^ (1)

σ_b_ = 629.3983 − 1.09679672 ∗ t + 0.00139 ∗ t^2^ + 0.00000217317795 ∗ t^3^ − 0.0000000042216372 ∗ t^4^ (2)

**Figure 17:**
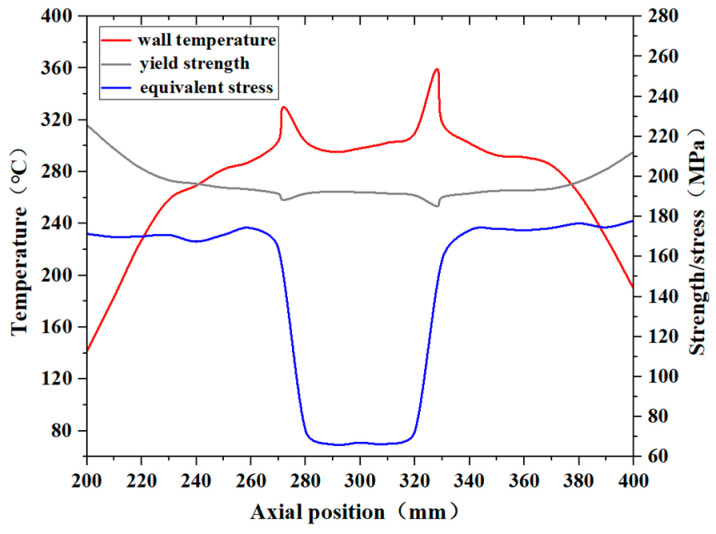
The equivalent stress, temperature, and yield strength distribution of the pipe wall at the corresponding temperature under the condition of 5.07 L/min.

The distributions of the equivalent stress, temperature, and yield strength at the corresponding temperatures were extracted for the pipe wall at the 150° circumferential position over the axial distance from 200 mm to 400 mm, and are presented in Figure 17. This region was characterized by high wall stress and elevated wall temperatures. The specific yield strength values at different temperatures were derived through polynomial fitting, using the data from the Materials Handbook. As illustrated in the figure, the middle section of the pipe, where the connection is located, has a thicker wall and a lower equivalent stress. Although the wall thickness is uniform on both sides of the pipe, the equivalent stress distribution is uneven due to the thermal stress caused by the temperature gradients and the applied constraints. The region of the distribution curve where the equivalent stress approaches the yield strength is located at approximately 350–370 mm in the axial direction, with a difference of about 20 MPa, indicating a higher risk of rupture under the conditions analyzed in this study.

**Figure 18:**
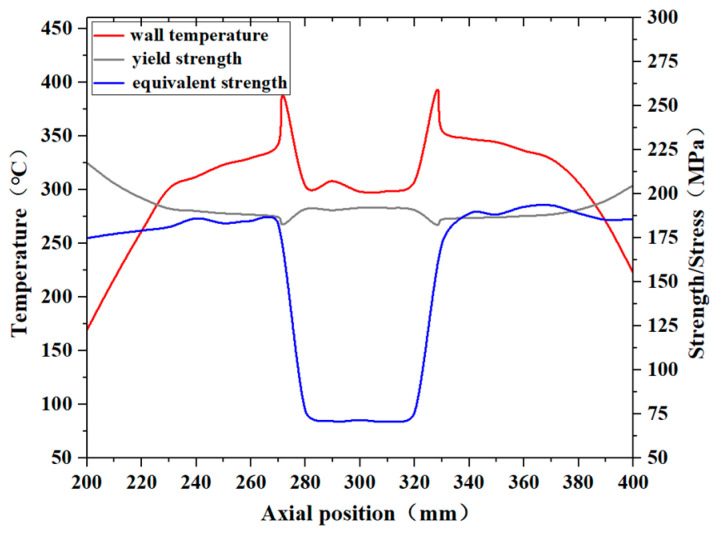
The equivalent stress, temperature and yield strength distribution of the pipe wall at the corresponding temperatures under the condition of 3.80 L/min.

Figure 18 presents the equivalent stress, temperature distribution, and material yield strength of the pipe wall at the 150° circumferential position when the kerosene inlet flow rate is 3.80 L/min. As shown in the figure, when the inlet flow rate decreases to 3.80 L/min, which is 75% of the flow rate specified in the fire test standard, the equivalent stress at the axial position between 340 mm and 380 mm exceeds the yield strength at the corresponding temperature, leading to permanent plastic deformation in this region. This occurred because the reduced inlet flow rate diminishes the heat transfer capacity of the kerosene, resulting in an increase in the pipe wall temperature and a reduction in the material’s yield strength. Furthermore, the rise in temperature gradients in all directions amplified the thermal stress, thereby increasing the equivalent stress on the wall. The location where the equivalent stress exceeded the yield strength the most is at the axial position of 370 mm.

**Figure 19:**
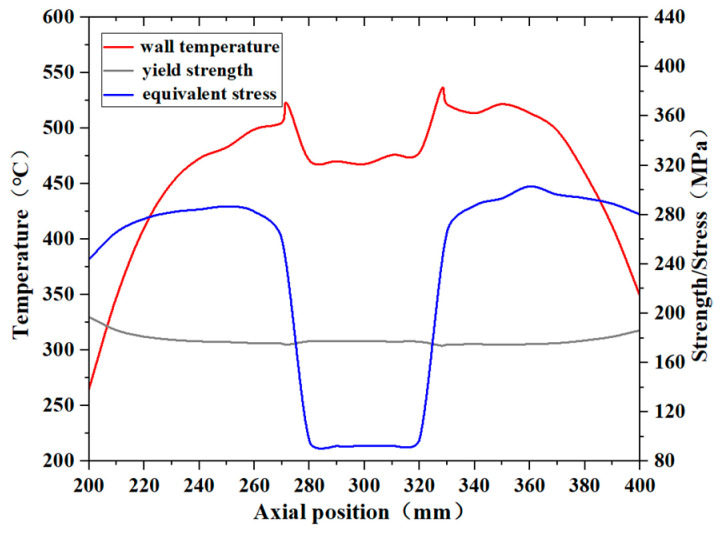
The equivalent stress, temperature, and yield strength distribution of the pipe wall at the corresponding temperature under the condition of 0.5 L/min.

As illustrated in Figure 19, when the kerosene inlet flow rate drops to 0.5 L/min, which is 10% of the flow rate specified in the fire test standard, the equivalent stress along the axial position from 200 mm to 400 mm exceeds the yield strength at the corresponding points. The maximum difference between the two occurs between 360 mm and 370 mm, with a difference of approximately 130 MPa, indicating that the pipe wall experiences significant plastic deformation. According to the Materials Handbook, the equivalent stress at this position also approaches the material’s tensile strength at the corresponding temperature, with a difference of about 110 MPa. Considering that the engine pipe is likely subjected to additional loads beyond the thermal loads, such as vibration, and that the deformation of the pipe wall may affect the oil flow and heat transfer (this study only considers the fluid’s impact on the pipe wall), the actual working environment of the pipe is significantly more challenging, and the risk of rupture is much higher.

From the above safety analysis, it is evident that as the kerosene flow rate decreases, the pipe wall stress increases, while the material strength diminishes, leading to a higher risk of fracture. Through a numerical simulation, this study finds that, under fire test conditions, the pipe wall located approximately 70 mm downstream from the center of the connection is at a particularly high risk of rupture. Therefore, in the design of aero-engine fuel and lubricating oil systems, measures should be implemented to enhance the fire resistance of this critical location.

## 5. Conclusions

In this paper, numerical simulations of flow–solid heat coupling for the aero-engine oil pipeline and its connecting section under different heating conditions were conducted based on standard documents. Subsequently, the safety of the pipe was analyzed by comparing the calculated stress results with the material strength of the pipe. The main conclusions are as follows:

1. Under the heating conditions specified in the fire test standard, the pipe wall temperature exhibited a three-layer stepped distribution along the axial direction, with higher temperatures in the middle and lower temperatures on both sides. The temperature rise between the first and second layers was primarily influenced by the lateral sweep of the hot flue gas, while the increase between the second and third layers was mainly due to the expansion of the heating surface area. The temperature distribution of the outer wall in the circumferential direction was also uneven, which generated thermal stress and, consequently, raised the risk of pipe wall rupture.
2. As the kerosene flow rate decreased, both the pipe wall temperature and the kerosene outlet temperature increased, while the heat transfer coefficient between the kerosene and the pipe wall exhibited an overall decline. This suggests a deterioration in the heat transfer efficiency. However, when the flow rate was decreased further, the effect of the changes in the thermophysical properties of the kerosene due to the temperature rise became significant. At this point, the heat transfer coefficient between the kerosene and the pipe wall no longer decreased significantly with the flow rate. The Reynolds and Prandtl numbers for the middle section of the pipe indicated significant changes in the flow characteristics and thermophysical properties of the kerosene, while the Nusselt number indicated that the calculated actual Nusselt number was greater than that obtained from the empirical formula. This suggests that the changes in the thermophysical properties of the supercritical kerosene significantly influenced its heat transfer behavior.
3. An increase in the temperature amplified the temperature gradient across the pipe wall, which led to a higher thermal stress and a reduction in the material’s yield strength, thereby raising the risk of a pipe wall rupture. Under the fire test conditions, when the kerosene inlet flow rate decreased to 75% of the standard flow rate, the local equivalent stress of the pipe wall exceeded the material’s yield strength at the corresponding temperature. When the flow rate further decreased to 10% of the standard, the equivalent stress at most locations on the pipe wall surpassed the yield strength. Given that the engine pipe was also subjected to additional loads, such as vibration, and that a pipe wall deformation can alter the flow and heat transfer conditions, the actual working environment of the pipeline was likely even more severe, with an elevated risk of rupture.
4. In this study, the pipe wall located approximately 70 mm downstream from the center of the connection along the flow direction was found to have a higher risk of rupture under the fire test conditions. Therefore, it is crucial to enhance the fire resistance of this area in the design of aero-engine fuel and lubricating oil systems.

## Figures and Tables

**Table 1:** Chemical composition of 1Cr18Ni9Ti.

Element	C	Mn	Si	Cr	Ni	S	P	Ti
Content, %	≤0.12	≤2.0	≤0.80	17.00~19.00	8.00~11.00	≤0.025	≤0.035	5 × (C% − 0.02%)~0.80%

**Table 2:** Thermal conductivity of 1Cr18Ni9Ti.

Temperature, °C	100	200	300	400	500	600	700	800	900
λ, W/(m·K)	16.3	17.6	18.8	20.5	21.8	23.5	24.7	26.4	28.5

**Table 3:** The kerosene inlet conditions.

Operating Condition Number	1	2	3	4	5	6
Pressure (MPa)	28	28	28	28	28	28
Temperature (K)	366.15	366.15	366.15	366.15	366.15	366.15
Flow rate (L/min)	5.07	3.80	2.54	1.27	0.50	0.25

## Data Availability

The original contributions presented in the study are included in the article, further inquiries can be directed to the corresponding author.
